# Quantum-Inspired Complex-Valued Language Models for Aspect-Based Sentiment Classification

**DOI:** 10.3390/e24050621

**Published:** 2022-04-29

**Authors:** Qin Zhao, Chenguang Hou, Ruifeng Xu

**Affiliations:** 1 School of Computer Science and Technology, Harbin Institute of Technology, Shenzhen 518055, China; a0086307@u.nus.edu; 2 Center for Remote Imaging, Sensing and Processing, National University of Singapore, Singapore 119076, Singapore; a0086303@u.nus.edu; 3 Peng Cheng Laboratory, Shenzhen 518066, China

**Keywords:** quantum language model, complexification, aspect-based sentiment analysis

## Abstract

Aiming at classifying the polarities over aspects, aspect-based sentiment analysis (ABSA) is a fine-grained task of sentiment analysis. The vector representations of current models are generally constrained to real values. Based on mathematical formulations of quantum theory, quantum language models have drawn increasing attention. Words in such models can be projected as physical particles in quantum systems, and naturally represented by representation-rich complex-valued vectors in a Hilbert Space, rather than real-valued ones. In this paper, the Hilbert Space representation for ABSA models is investigated and the complexification of three strong real-valued baselines are constructed. Experimental results demonstrate the effectiveness of complexification and the outperformance of our complex-valued models, illustrating that the complex-valued embedding can carry additional information beyond the real embedding. Especially, a complex-valued RoBERTa model outperforms or approaches the previous state-of-the-art on three standard benchmarking datasets.

## 1. Introduction

Aspect-Based Sentiment Analysis (ABSA) is a fine-grained sentiment analysis task, whose aim is to classify the sentiment polarities of a sentence over one or more aspects [1,2,3,4,5]. The fundamental sentiment elements involved in the ABSA tasks are aspect category, aspect term, opinion term, and sentiment polarity. Aspect category defines into which category an aspect term should fall. For example, in the restaurant domain, “Food” and “Service” are the aspect categories. Aspect term is the opinion target shown in the given text. Opinion term expresses one’s sentiment towards the aspect term. Sentiment polarity depicts the orientation of the sentiment on an aspect category or an aspect term which usually be positive, negative, or neutral. For instance, given a sentence “the food was great and the service was severely slow”, the first aspect term is “food” which belongs to category “Food”, and the opinion term is “great”. Therefore, the sentiment polarity for aspect term “food” and aspect category “Food” is positive. The second aspect term is “service” which falls into category “Service”, and the opinion term is “slow”. Hence, the sentiment polarity for aspect term “service” and aspect category “Service” is negative. Generally, ABSA consists of two subtasks: aspect extraction (AE) and aspect-level sentiment classification (ALSC). Our paper only focuses on ALSC, which is to predict the exact sentiment polarities of different aspect terms in their context, instead of classifying the overall sentiment polarity on a sentence level or document level. That is, our task is to classify the sentiment polarities of aspect terms, such as “food” and “service” mentioned in the above example, in a given context. Such sentiment polarity classification over aspects can be used to better investigate the fine-grained emotional tendency in reviews and hence can provide more accurate recommendations to decision makers.

Previously, to avoid designing hand-crafted features, a large number of deep-learning-based neural network models have been proposed to solve ALSC tasks, such as RNN-based models [6,7,8,9], CNN-based models [10,11]. In order to better classify aspect-level sentiment, target information has also been taken into account when constructing neural networks. Attention mechanism, which has been proven effective in image recognition, machine translation, sentence summarization, etc. [12,13,14], has also been introduced to ALSC tasks [9], with the attention weight of different words dynamically calculated considering the relationship between words and aspects. Recently, dominating across various NLP tasks, pre-trained transformer-based models have also received a lot of attention in ALSC tasks. BERT and RoBERTa-based models achieve outstanding success on various ABSA benchmark datasets [15,16,17,18,19].

However, the vector representations used in most of those models are constrained to real values. As a fundamental concept widely applied in various fields, such as signal processing, quantum physics, and medical image processing, complex-valued vectors are composed of a pair of correlated real and imaginary vectors in orthogonal dimensions. Due to the richer representational capacity of complex-valued vectors, complexified neural networks have already been applied in numerous fields, for instance, signal processing [20,21], computer vision [22], and natural language processing [23]. Multi-dimensional real-valued input vectors can be naturally expressed as complex-valued ones when mapped into frequency or wavevector space from a quantum theory perspective.

Inherently featuring compatibility with complex-valued vectors, quantum language models (QLMs) inspired by quantum theory are drawing more and more attention. In quantum language models, every word is naturally represented as an observed state in a quantum system in the Hilbert Space, and is represented by a superposition of sememes [24,25]. From such quantum perspective view, one can build neural network in a more principled approach by drawing analogies between quantum operators and neural network calculation, and applying the methods developed by quantum physicistis to make up for the lack of interpretability of neural networks in NLP. Based on such background, Li et al. [25] constructed a complex-valued network for question answering task, and Zhao et al. [26] proposed a quantum expectation value-based language model.

Motivated by the excellent work in quantum language models and their compatibility with complex-valued vectors, we investigate employing the complex-valued representations from the mathematical framework of quantum physics to solve ABSA tasks. To this end, complex-valued neural networks are built by introducing a semantic Hilbert space, where a word of a ABSA model is viewed as a physical state, encoded as a complex-valued vector. To benchmark the resulted models’ performance, each model is evaluated against its corresponding real-valued baseline. In this study, we construct the complexification of three strong real baselines, namely, complex-valued LSTM model, complex-valued attention-based LSTM model, and complex-valued BERT/RoBERTa model. From mathematical perspective, the complexification of a real vector space *V* is defined by taking the tensor product of *V* with a complex number. Here, the complexification of real-valued baseline is following the similar operation. Noteworthily, by setting the imaginary parts of complex-valued vectors to zero, the complex-valued models just shrink to their real-valued equivalent.

Experiment results evaluated on three benchmarking datasets, namely, Twitter, Restaurant 14, Laptop 14, demonstrate the outperformance of our complex-valued models, and illustrate that the complex-valued embedding could carry additional information beyond the real embedding. Specifically, both of the complex-valued versions of LSTM model and attention-based LSTM model outperform the original ones. Meanwhile, the complex-valued RoBERTa model outperforms or nears to the previous SOTA on the three standard benchmarks. The results imply that the complexification extended from quantum physical particle representation has a potential to be encapsulated to general language models in ABSA.

## 2. Related Work

In this section, the related studies on ALSC tasks and QLMs will be introduced.

To solve ALSC tasks, recurrent neural networks and convolutional neural networks are among the most commonly used deep neural network architectures. Incorporating information from target words, Tang et al. [8] first proposed two single-directional LSTMs (TD-LSTMs), which handle the left and right context of the target word independently. Afterwards, attention mechanism was adopted in the ALSC task. Designed by Tang et al. [27], MemNet uses a multi-hop attention to reveal the importance of each context word with respect to aspect targets. Wang et al. [9] designed ATAE-LSTM which takes the concatenation of aspect and context word embedding as an input and applies the attention mechanism to dynamically computing attention weight. Utilizing multiple-attention mechanism with memory layers, Recurrent Attention on Memory (RAM) released by Chen et al. [28] is a bidirectional LSTM to obtain global semantic features.

Recently, transformer-based models which have dominated across various NLP tasks, have also drawn much attention to ALSC task. Song et al. [15] proposed BERT-SPC which is a pure BERT text pair classification model and achieves outstanding performance. With a pre-trained BERT supplying input word embeddings, they simultaneously designed an Attentional Encoder Network (AEN), which could derive semantic word-context interactions using the attention mechanism. Yang et al. [16] proposed a local context focus (LCF) mechanism based on multi-head self-attention. BERT-ADA shows that the pre-trained BERT adapted only to specific tasks can be further improved through a fine-tuning process on a task-related corpus [17].

The first QLM is proposed by Sordoni, Nie and Bengio [29] in Information Retrieval (IR), by simplifying the Hilbert space to a real space. Inspired by their work, a wide range of research on quantum language models has been studied [24,30]. Limited to real space, Zhang et al. [24] proposed an end-to-end Neural Network-based Quantum-like Language Model (NNQLM) to solve question answering task. In the model, every word is viewed as a pure quantum state in the system, and question and answer sentences are respectively characterized by their corresponding density matrices. Adopting tensor product to describe the interaction among an entire word sequence, Zhang et al. [31] built a Quantum Many-body Wave Function-inspired language model with only real-valued embeddings.

It is noteworthy that the vector representations used in the aforementioned models are only constrained to real-value neural networks. Especially for QLMs, they oversimplify the Hilbert space as a real sub-space. Therefore, complex-valued quantum language models were proposed afterwards, by representing the physical state of a quantum system properly as a complex-valued function. In question-answering tasks, the complex-valued quantum language models have been successfully applied, and demonstrates that a word representation in a complex form could feature more information beyond the real one, supported by the additional imaginary part. Among these models, Li et al. [25] built a Complex-valued Network for Matching (CNM), in which each word is encoded in a polar coordinate system as a complex-valued vector, with its length and direction representing the relative word weight and a superposition, respectively. Applying complex embedding, Zhao et al. [26] proposed a quantum expectation value-based language model. Under this framework, a language model can have excellent interpretability and performance. Li et al. also investigated complex-valued neural network in video sentiment analysis tasks and their model achieves comparable results with state-of-the-art models [32].

However, to the authors’ knowledge, the complex-valued framework has not given rise to any applications in ABSA. Therefore, inspired by the exciting work in quantum language models and their compatibility with complex-valued vectors, we study the application of complex-valued representation from the mathematical framework of quantum physics to ABSA tasks.

## 3. Background

In quantum probability [33], the probabilistic space is naturally represented in a Hilbert space, denoted as $H^{n}$.

In this section, we briefly introduce some basic concepts in quantum probability theory. As a generalization of the classical probability theory, quantum probability theory provides a mathematical interpretation on physics phenomena involving quantum particles, such as electrons and photons. The probabilistic space describing the wave-function of particles is represented in a Hilbert space, denoted as $H^{n}$, which is an infinite-dimensional inner product space over complex numbers [33]. In this Hilbert space, a complex-valued unit vector $\vec{u}\in H^{n}$ and its Hermitian conjugate ${\vec{u}}^{†}$ can be expressed as *ket*|u〉 and a *bra*〈u| respectively, following Dirac notation. The inner product of two unit vectors |u〉 and |v〉 is written as 〈u|v〉. Given an orthonormal bases $\{|e_{i}\rangle {\}}_{i=1}^{n}$ for $H^{n}$, an arbitrary vector |u〉 can be expanded as a linear combination of basis vectors as follows:

(1) $$|u\rangle =\sum_{i=1}^{n}u_{i}|e_{i}\rangle ,$$

where $u_{i}$ is the probability amplitude along $|e_{i}\rangle$ and satisfies $\sum_{i}{\left|u_{i}\right|}^{2}=1$.

## 4. Complex-Valued Language Models

Inspired by the quantum language models, a word is viewed as a physical observable in a quantum system, and it is represented by a complex-valued vector. Under this scope, three QLMs, which all take complex-valued embeddings as inputs, are furthermore constructed as the complexification of three strong real-valued baselines, namely complex-valued LSTM models, complex-valued attention-based LSTM model, and complex-valued BERT/RoBERTa model. The resulted three complex-valued models are compared with the corresponding real-valued ones to benchmark their performance. Via the comparison, we hope to see that the imaginary part of embedding can carry additional information beyond the real part and further emphasize the importance of introducing quantum language models. First, we would like to make a clarification. In our paper, three complex-valued language models are built based on three typical real baselines. However, in addition to the chosen ones, there are still a large number of different types of neural networks for language tasks. We hope the three types of models can shed light on the effect of complex-valued structure on improving a model’s performance, and show the possibility to further explore the structure in other types of neural networks.

In this section, we first introduce complexification of a real vector space, the procedure to encode a word as a complex-valued vector, and then present the approach to construct the complexification of the three real baselines.

### 4.1. Complexification of a Real Vector Space

If *V* is a real vector space, the complexification of *V* is defined by taking the tensor product of *V* with complex numbers *C* [34,35]:

(2) $$V^{C}=V⊗C.$$

Alternatively, rather then using tensor products, the complexification of a real space can be defined as follows:

(3) $$V^{C}=V⊕iV.$$

Therefore, every vector $v_{c}$ in $V^{C}$ can be written in the form

(4) $$v_{c}=v_{1}⊗1+v_{2}⊗i,v_{1}\in V,v_{2}\in V,$$

where $v_{1}$ and $v_{2}$ are vectors in *V*. Generally, one can drop the tensor product symbol and just write

(5) $$v_{c}=v_{1}+iv_{2}.$$

### 4.2. Complex-Valued Word Embedding

In quantum language models, the Hilbert space is the mathematical foundation of physical events studied. Based on this background, our proposed models are constructed.

Since a quantum state is usually complex-valued, we therefore introduce the Semantic Hilbert Space $H^{n}$ on a complex vector space $C^{n}$, spanned by a set of orthogonal bases states $\{|e_{j}\rangle {\}}_{j=1}^{n}$. $|e_{j}\rangle$ represents a sememe which is the minimum semantic unit of word meanings in language universals [36], and is an one-hot vector with only the *j*-th element in $|e_{j}\rangle$ being one while all the other elements being zero. A word *w* is viewed as a physical state in such semantic Hilbert space, and hence can be represented as a superposition of sememes, written as follows:

(6) $$\begin{array}{c} |w\rangle =\sum_{j=1}^{n}\left(w_{rj}+iw_{mj}\right)|e_{j}\rangle =|w_{r}\rangle +i|w_{i}\rangle , \end{array}$$

where $|w_{r}\rangle =\sum_{j=1}^{n}w_{rj}|e_{j}\rangle$ and $|w_{i}\rangle =\sum_{j=1}^{n}w_{mj}|e_{j}\rangle$ are the real part and imaginary part of the state |w〉, respectively. ${\left\{w_{rj}\right\}}_{j=1}^{n}$ and ${\left\{w_{mj}\right\}}_{j=1}^{n}$ are the real part and imaginary part of probability amplitudes along sememes.

To encode the complex-valued word embedding, we follow the method of the complexification of a real vector as Equation (5). In the conventional standard neural network, a word is encoded as a vector containing rich semantic information in word embedding lookup table. Following the same encoding convention, we first choose a real space *E* formed by vectors in the lookup table, and then consider as the complexification of *E* as the semantic Hilbert space. Therefore, an arbitrary word |w〉 is embedding as:

(7) $$\begin{array}{c} \left|w\rangle =\right|w_{r}\rangle +i|w_{i}\rangle ,w_{r}\in E,w_{i}\in E, \end{array}$$

where both $|w_{r}\rangle$ and $|w_{i}\rangle$ are vectors in the real word embedding space *E*. Know that a word *w* in conventional neural network is embedded using the lookup table as vector *X* belonging to *E*. To carry useful semantic information, we set $|w_{r}\rangle$ and $|w_{i}\rangle$ as a linear transformation of *X*. Then we have

(8) $$\begin{array}{c} w_{r}=U_{r}\cdot X^{T}, \end{array}$$

(9) $$\begin{array}{c} w_{i}=U_{m}\cdot X^{T}. \end{array}$$

Here, $U_{r}$ and $U_{m}$ are linear transformation parameters.

We can utilize a L2 normalization to restrict every word *w* to a unit length as follows:

(10) $$|w\rangle =\frac{\vec{w}}{\|\vec{w}\|},$$

where $\|\vec{w}\|$ is the L2-norm of $\vec{w}$.

### 4.3. Complex-Valued Models

#### 4.3.1. Complex-Valued LSTM Model

**Figure 1:**
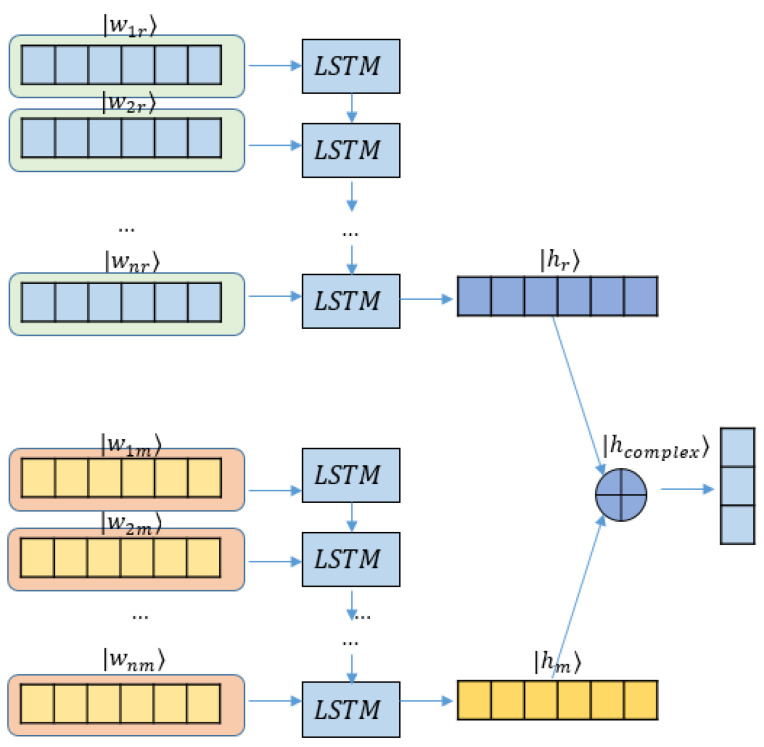
Complex-valued LSTM model (C-LSTM).

As a typical recurrent neural network, LSTM [37] which can learn context information and abstract low-dimensional representations of words and sentences, has achieved great success in various NLP tasks. To assess the effectiveness of the complexification of neural networks in ABSA, we first construct a complex-valued LSTM model (C-LSTM), whose overall architecture is shown in Figure 1.

In the modified model, each word is embedded as a complex-valued vector following Equation (8). Since word embedding matrix *E* is trained on a large-scale corpus rich in semantic information, we assume that both of the real and imaginary probability amplitudes ${\left\{w_{rj}\right\}}_{j=1}^{n}$ and ${\left\{w_{mj}\right\}}_{j=1}^{n}$ are linear with the word vector from *E*. Meanwhile the embedding matrix *E* is a |V|×d square matrix in a real space, viz. $E\in R^{|V|\times d}$, where |V| is the size of the vocabulary and *d* is the dimension of the word embedding.

To minimize the impact of complexification on the LSTM backbone and highlight the effect of imaginary parts on carrying extra semantic information, two separate LSTMs are utilized to generate the real and imaginary parts of a hidden vector individually, instead of blending the complex pipe inside the original LSTM workflow.

For the real part, we have

(11) $$\begin{array}{cc} w_{r} & =U_{r}\cdot X^{T}, \end{array}$$

(12) $$\begin{array}{cc} f_{tr} & =\sigma (W_{fr}\cdot w_{r}+U_{fr}h_{(t-1)r}+b_{fr}), \end{array}$$

(13) $$\begin{array}{cc} i_{tr} & =\sigma (W_{ir}\cdot w_{r}+U_{ir}h_{(t-1)r}+b_{ir}), \end{array}$$

(14) $$\begin{array}{cc} o_{tr} & =\sigma (W_{or}\cdot w_{r}+U_{or}h_{(t-1)r}+b_{or}), \end{array}$$

(15) $$\begin{array}{cc} c_{tr} & =f_{tr}⊙c_{(t-1)r}+i_{tr}⊙\tanh\left(W_{cr}\cdot w_{r}+b_{cr}\right), \end{array}$$

(16) $$\begin{array}{cc} h_{tr} & =o_{tr}⊙\tanh\left(c_{tr}\right), \end{array}$$

where *X* is the vector from the lookup embedding matrix. As shown in (7), by performing a linear transformation via $U_{r}$, we obtain the real embedding vector $w_{r}$. The subscript *r* for vectors refers to vectors for the real part. $f_{tr}$ is the forget gate’s activation vector. $i_{tr}$ denotes the input and update gate’s activation vector. $o_{tr}$ is the output gate’s activation vector. $c_{tr}$ is the cell state vector. $h_{tr}$ represents hidden state vectors. $W_{fr}\in R^{h\times d}$, $W_{ir}\in R^{h\times d}$, $W_{or}\in R^{h\times d}$, $W_{cr}\in R^{h\times d}$, $U_{fr}\in R^{h\times h}$, $U_{ir}\in R^{h\times h}$, $U_{or}\in R^{h\times h}$ are the weight matrices and $b_{fr}\in R^{h}$, $b_{ir}\in R^{h}$, $b_{or}\in R^{h}$, $b_{cr}\in R^{h}$ are biases of LSTM to be trained. Here, *d* and *h* refer to the dimension of the real embedding vector and number of hidden units, respectively. σ is the sigmoid function and ⊙ indicates element-wise multiplication.

On the imaginary part, the similar operations are performed:

(17) $$\begin{array}{cc} w_{m} & =U_{m}\cdot X^{T}, \end{array}$$

(18) $$\begin{array}{cc} f_{tm} & =\sigma (W_{fm}\cdot w_{m}+U_{fm}h_{(t-1)m}+b_{fm}), \end{array}$$

(19) $$\begin{array}{cc} i_{tm} & =\sigma (W_{im}\cdot w_{m}+U_{im}h_{(t-1)m}+b_{im}), \end{array}$$

(20) $$\begin{array}{cc} o_{tm} & =\sigma (W_{om}\cdot w_{m}+U_{om}h_{(t-1)m}+b_{om}), \end{array}$$

(21) $$\begin{array}{cc} c_{tm} & =f_{tm}⊙c_{(t-1)m}+i_{tm}⊙\tanh\left(W_{cm}\cdot w_{m}+b_{cm}\right), \end{array}$$

(22) $$\begin{array}{cc} h_{tm} & =o_{tm}⊙\tanh\left(c_{tm}\right). \end{array}$$

$w_{m}$ is the imaginary embedding vector via a linear transformation matrix $U_{m}$. Subscript *m* is used to indicate a vector for the imaginary part. $f_{tm}$, $i_{tm}$ and $o_{tm}$ are the forget gate’s activation vector, the input and update gate’s activation vector and the output gate’s activation vector, respectively. Considering the real part LSTM and imaginary LSTM having the same size of word embedding vector and hidden units, weight matrices $W_{fm}$, $W_{im}$, $W_{om}$, and $W_{cm}$ have the shape h×d. $U_{fm}$, $U_{im}$ and $U_{om}$ have the shape h×h. Biases $b_{fm}$, $b_{im}$, $b_{om}$, and $b_{cm}$ are *h*-dimensional vectors.

Therefore, the real and imaginary LSTMs produce the final output hidden state $h_{nr}$ and $h_{nm}$, respectively. Omitting *n* subscript, they join together to form a complex hidden state $h_{complex}$ as follows:

(23) $$h_{complex}=h_{r}+ih_{m}.$$

The complex state vector is then fed to a dense layer to perform the last polarity classification.

#### 4.3.2. Complex-Valued Attention-Based LSTM Model

**Figure 2:**
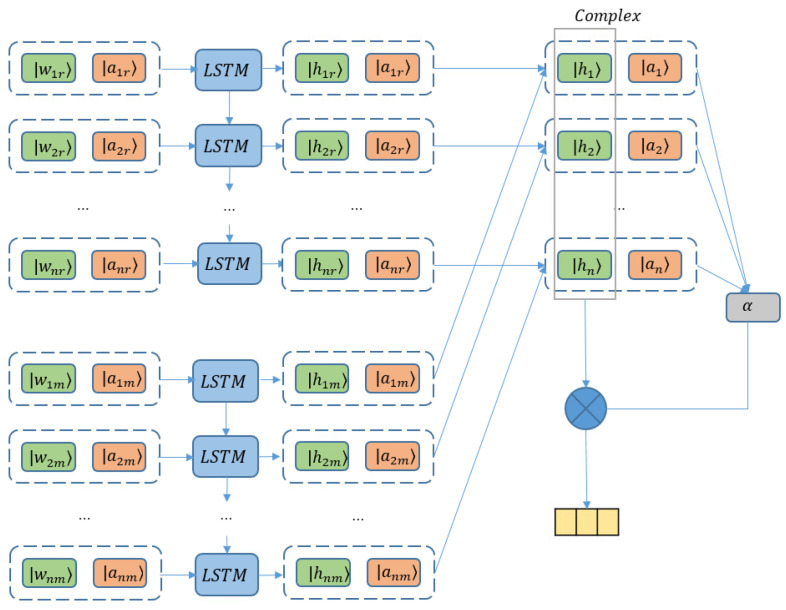
Complex-valued attention-based LSTM model (C-ATAE-LSTM).

The attention mechanism has been demonstrated to be effective to obtain better performance in various fields, for example, image recognition [12], machine translation [13], sentence summarization [38], and so on. By enhancing the important portions and diminishing the irrelevant words of a sentence with weights upon the aspects concerned, attention also provides effectiveness gain on aspect-based sentiment classification. As a typical attention-based LSTM model, ATAE-LSTM has been treated as a standard baseline [9]. Therefore, to manifest the influence of the complex-valued scheme on an attention model, we construct the complexification of ATAE-LSTM (C-ATAE-LSTM), with its sketch illustrated in Figure 2.

The real and imaginary parts of word embeddings are obtained in the same way with Equation (11). Then concatenated with complex-valued aspect information, word representations are fed to LSTM layers as follows:

(24) $$\begin{array}{cc} \lambda_{\alpha } & =\left[\begin{array}{c} a_{\alpha }\\ w_{\alpha } \end{array}\right],\alpha \in \left\{r,m\right\}, \end{array}$$

(25) $$\begin{array}{cc} w_{\alpha } & =U_{r}\cdot \lambda_{\alpha }^{T},\alpha \in \left\{r,m\right\}, \end{array}$$

(26) $$\begin{array}{cc} f_{t\alpha } & =\sigma \left(W_{f\alpha }\cdot w_{\alpha }+U_{f\alpha }h_{(t-1)\alpha }+b_{f\alpha }\right),\alpha \in \left\{r,m\right\}, \end{array}$$

(27) $$\begin{array}{cc} i_{t\alpha } & =\sigma \left(W_{i\alpha }\cdot w_{\alpha }+U_{i\alpha }h_{(t-1)\alpha }+b_{i\alpha }\right),\alpha \in \left\{r,m\right\}, \end{array}$$

(28) $$\begin{array}{cc} o_{t\alpha } & =\sigma \left(W_{o\alpha }\cdot w_{\alpha }+U_{o\alpha }h_{(t-1)\alpha }+b_{o\alpha }\right),\alpha \in \left\{r,m\right\}, \end{array}$$

(29) $$\begin{array}{cc} c_{t\alpha } & =f_{t\alpha }⊙c_{(t-1)\alpha }+i_{t\alpha }⊙\tanh\left(W_{c\alpha }\cdot w_{\alpha }+b_{c\alpha }\right),\alpha \in \left\{r,m\right\}, \end{array}$$

(30) $$\begin{array}{cc} h_{t\alpha } & =o_{t\alpha }⊙\tanh\left(c_{t\alpha }\right),\alpha \in \left\{r,m\right\}. \end{array}$$

Here, α takes value from {r,m}. *r* and *m* denote the real and imaginary components, respectively. Therefore, when α=r, $a_{\alpha }$ is $a_{r}$, which is the real embedding of aspect; when α=m, $a_{\alpha }$ is $a_{m}$, which is the imaginary embedding of aspect. $w_{r}$ and $w_{m}$ are the real and imaginary embeddings for a word; $\lambda_{\alpha }$ is the concatenation of $w_{\alpha }$ and $a_{\alpha }$; $W_{f\alpha }\in R^{h\times d}$, $W_{i\alpha }\in R^{h\times d}$, $W_{o\alpha }\in R^{h\times d}$, $W_{c\alpha }\in R^{h\times d}$, $U_{f\alpha }\in R^{h\times h}$, $U_{i\alpha }\in R^{h\times h}$, $U_{o\alpha }\in R^{h\times h}$ are the weight matrices. $b_{f\alpha }\in R^{h}$, $b_{i\alpha }\in R^{h}$, $b_{o\alpha }\in R^{h}$, $b_{c\alpha }\in R^{h}$ are biases of LSTM to be trained. *d* and *h* refer to the dimension of the real embedding vector and the number of hidden units, respectively. After concatenating all the output vectors of hidden states, we write the final output of the hidden layer as follows:

(31) $$\begin{array}{cc} H_{r} & =Concat\{h_{1r},h_{2r},\cdots ,h_{nr}\}, \end{array}$$

(32) $$\begin{array}{cc} H_{m} & =Concat\{h_{1m},h_{2m},\cdots ,h_{nm}\}, \end{array}$$

(33) $$\begin{array}{cc} H & =H_{r}+iH_{m}. \end{array}$$

With the contribution of the real part output $H_{r}$ and imaginary output $H_{m}$, $H\in C^{d\times N}$ is the matrix consisting of complex-valued hidden vectors, where *d* is the dimension of hidden vectors and *N* is the length of the given sentence. Now, we apply the attention mechanism on the complex-valued hidden vectors.

(34) $$\begin{array}{cc} M & =\tanh(W_{h+a}\left[\begin{array}{c} H\\ a \end{array}\right]), \end{array}$$

(35) $$\begin{array}{cc} \alpha & =softmax(\left(u^{T}M\right).real), \end{array}$$

(36) $$\begin{array}{cc} H_{output} & =H\alpha^{T}, \end{array}$$

where $W_{h+a}$ and *u* are trainable complex-valued transformation matrices. As the final attention weight can be viewed as the probability of $H_{output}$ on the corresponding hidden vector which is real, we project $u^{T}M$ to a real number.

#### 4.3.3. Complex-Valued BERT/RoBERTa Model

**Figure 3:**
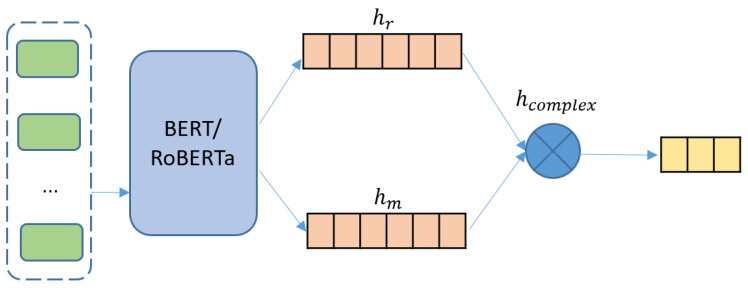
Complex-valued BERT/RoBERTa model (C-BERT/C-RoBERTa).

As a state-of-the-art pre-trained language model, transformer-based BERT (Bidirectional Encoder Representations from Transformers) has achieved tremendous success in many natural language processing tasks [39], and has become a fundamental component in various models. To investigate the influence of complex-valued representation on such model, we study the complexification of pure BERT and RoBERTa models (C-BERT and C-RoBERTa) [15,40], whose sketch is shown in Figure 3.

Here, the input sequence of C-BERT is formed as “[CLS] + context + [SEP]” and “[CLS] + target + [SEP]” following BERT’s standard. Similar to the former construction of complex-valued word embeddings, we view the real and imaginary context representations as a linear function of the dropout of BERT’s output vectors as follows:

(37) $$\begin{array}{cc} X & =BERT(input), \end{array}$$

(38) $$\begin{array}{cc} h_{\alpha } & =U_{\alpha }X,\alpha \in \left\{r,m\right\}, \end{array}$$

(39) $$\begin{array}{cc} h_{complex} & =h_{i}+ih_{m}, \end{array}$$

where *X* is the direct output vector from BERT possessing the context’s representation. Context, as a collection of words, can be viewed as a superposition of word states, and hence is also a physical state in the quantum system. After obtaining the fundamental semantic and context information *X* for a sentence, we assume that the real and imaginary part, $h_{r}$ and $h_{m}$, of a sentence is a linear transformation of *X*, following the same method to construct a word representation.

### 4.4. Model Training

The final sentence representations, derived from the above three models, are forwarded into a dense layer to produce the polarity classification. We train the three models independently. For every model, the final output is projected to the probabilities over the three classes (positive, neutral, negative) via a softmax layer. We denote the predicted sentiment distribution as $\hat{y}$ and the ground truth label as *y*. The complete models are trained in an end-to-end way with cross-entropy as the loss function. The training goal is to minimize the negative cross entropy loss as follows:

(40) $$L=-\sum_{i}\sum_{i}y_{i}^{j}\log{\hat{y}}_{i}^{j}+{\lambda ||\theta ||}^{2},$$

where *i* is the index of sentence, *j* is the index of class, λ is the coefficient of the $L_{2}$ regularization term, and θ is the parameter set.

## 5. Experiment

### 5.1. Experimental Setup

As the target task of our study, ALSC is to determine whether the polarity of every aspect term is positive, negative, or neutral, for a given set of aspect terms in a sentence. The experiments are conducted on three widely used benchmarking datasets for ABSA, whose statistics are summarized in Table 1:

- Twitter is a dataset gathered by Dong et al. [41];
- Restaurant and Laptop are downloaded from SemEval 2014 task 4 [4], which contains sentiment reviews for restaurant and laptop domains.

These datasets are labeled with three sentiment polarities: positive, neutral, and negative. Similar to the previous works [7], samples with conflicting polarities and “NULL” aspects in datasets are removed. It is worth mentioning that there are also other datasets for ABSA tasks. However, only the above three benchmarking datasets are widely used in the ALSC task, for which plenty of ALSC models are implemented [2]. In order to have a better comparison with those models, we also evaluate our models on those benchmarking datasets.

### 5.2. Baselines for Comparison

A comprehensive comparison with a wide range of models is conducted, in order to comprehensively evaluate our models’ performance. We compare our models with basic RNN baselines including LSTM, TD-LSTM, and ATAE-LSTM, followed by typical transformer-based BERT/RoBERTa models.

- LSTM [37]: it is a standard LSTM.
- TD-LSTM [8]: it is a target-dependent LSTM learning context and target information.
- ATAE-LSTM [9]: to carry aspect information, an aspect vector is concatenated to each of word embedding vectors. An attention mechanism is used to construct a sentence’s representation against different aspects.
- BERT-SPC [15]: it feeds sequences in the form of “[CLS] + context + [SEP] + target + [SEP]” into a basic BERT model for the sentence pair classification task.
- AEN-BERT [15]: it is an attentional encoder network based on the pre-trained BERT model, which draws hidden states and semantic interactions between target and context words.
- RoBERTa-MLP [40]: it is a pure RoBERTa model.
- BERT-ADA [17]: it is a domain-adapted BERT-based model finetuned on a task-related context.
- LCF-ATEPC [16]: it is a multi-task learning model for AE and ALSC, based on BERT-SPC model and local context focus mechanism, and is the state-of-the-art model on the Restaurant dataset.

It is worth mentioning that the baselines chosen for comparison are all published well-known classical neural networks. The reason why we do not compare our model with other quantum language models is that this work is the first attempt of quantum-inspired complex-valued model in ABSA field and hence there is a lack of information on other QLMs’ performance. Moreover, unlike other models such as CNM which introduces a complex-valued structure for a specific task [25], we focus more on the investigation of the complexification framework and its performance over the corresponding real baselines. Therefore, the comparison is mainly performed on its real counterparts.

### 5.3. Implementation Details

Comparing with a trainable word embedding, we find that a fixed embedding can lead to a better performance. Therefore, word embeddings in our model do not get updated in the learning process. For C-LSTM and C-ATAE-LSTM, word vectors, aspects and hidden states are 300-dimensional and complex-valued. For all word vectors and aspect embeddings, the real part and imaginary part are both initialized by Glove [42] with dimension 300, which are pre-trained on an unlabeled corpus. The dimension of the LSTM hidden complex vector is set to 300. For the C-BERT and C-RoBERTa models, the embedding dimension is 768. Hidden states are 300-dimensional complex-valued vectors. We adopt the Adam optimizer with the learning rate among $\left[1\times 10^{-5},2\times 10^{-5},1\times 10^{-4}\right]$ and choose the batch size to be 16.

### 5.4. Experimental Result

Table 2, Table 3 and Table 4 show the experimental results for the Twitter, Restaurant, and Laptop datasets, respectively. From the results, we can draw a conclusion that the three types of complex-valued models for ABSA achieve better performance than the corresponding real ones. Therefore, a complex-valued structure indeed can carry more semantic information, and hence has an ability to improve a real counterpart’s performance.

Specifically, compared with LSTM, C-LSTM works better on the Restaurant and Laptop datasets, with a higher accuracy and a better F1 value. It also outperforms TD-LSTM on Restaurant and Laptop datasets. This means that with an extra imaginary LSTM added, C-LSTM can carry additional information beyond the real embedding. When attention is added to an LSTM model, the complex-valued attention-based LSTM model outperforms ATAE-LSTM and achieves the best results within all of the chosen RNN baselines. The results where C-ATAE-LSTM exceeds the performance of C-LSTM show that the attention mechanism can improve a complex-valued model, and the better performance of C-ATAE-LSTM over ATAE-LSTM means that an attention mechanism can also benefit from the complex-valued framework. The results of C-BERT and C-RoBERTa models manifest that complex-valued representations can also improve a transformer-based model. Especially, the complex-valued RoBERTa model achieves the state-of-the-art performance on the Twitter and Laptop datasets. With comparisons ranging from standard RNN baselines to transformer-based models, the results imply that the quantum-inspired complexification framework has potential to be encapsulated to general language models in ABSA task and achieves better performance for predicting aspect-based sentiment. Moreover, since our complex-valued models are all quantum-inspired models, our results can also highlight the importance of quantum language models, which have a more fundamental theoretic background and also have an ability to improve the performance of traditional language models, due to their more complicated structures.

## 6. Discussion

To explicitly investigate the impact of our quantum-inspired complex-valued representation, we conduct several comparison studies.

### 6.1. Comparison with Random Imaginary Embedding

The comparison with the corresponding real baselines has already demonstrated that the complex-valued embedding could carry additional information beyond the real embedding. We encode the imaginary embedding as a linear function of sememes in the above models. To investigate the influence of such embedding, we build the ablation model where the imaginary embedding is trainable and initialized with a random normal distribution. The ablation models are named with the corresponding complex-valued models with suffix “-random”, as shown in Table 2, Table 3 and Table 4.

Experiment results in Table 2, Table 3 and Table 4 show that the disordered imaginary part will greatly diminish the performance of models, which is even much weaker than that of the real baselines. This further demonstrates that a semantic related embedding, free from harmful noise introduced by a random input embedding, can improve a model’s performance. Therefore, this further demonstrates in the following section that preserving the correlation information comprehended in the relative ratio between different embedding dimensions is important.

### 6.2. Hilbert Space Represented in Polar Coordinate System

Our semantic Hilbert Space is represented in a Euclidean coordinate system, where an arbitrary word can be decomposed as in Equation (8). A word in a quantum language model is treated as a physical state in a quantum system, whose physical property should be independent of the chosen coordinate system. To explore whether our complex-valued model’s performance depends on a specifically chosen coordinate system, we investigate another commonly used coordinate system, namely, a polar coordinate system, which is widely applied to representing the Hilbert Space $H^{n}$. Using a polar system, a word is expanded as:

(41) $$|w\rangle =\sum_{j=1}^{n}r_{j}e^{i\phi_{j}}|e_{j}\rangle ,$$

where $r_{j}$ is a non-negative real-valued amplitude of the state |w〉 along the radius direction, satisfying $\sum_{j=1}^{n}r_{j}^{2}=1$, and $\phi_{j}\in \left[-\pi ,\pi \right]$ is the corresponding phase of the state |w〉 in the polar coordinate system [25]. We initialize the $r_{j}$ with the 300-dimension Glove vectors, and all ${\left\{\phi_{j}\right\}}_{j=1}^{n}$ are chosen be to the same within [−π,π]. It is noteworthy that $\phi_{j}$ share a same value ϕ, which is independent of *j*, to preserve the correlation information comprehended in the relative ratio between different dimensions. Otherwise, uncorrelated random rotation will scarify the model performance by diminishing meaningful knowledge in word embedding, which is not trainable in our cases. For C-BERT model and C-RoBERTa model, we perform similar operations for the complex-valued context representation.

The results in Table 2, Table 3 and Table 4 show that the performance of all models in such a coordinate system is comparable to those in a Euclidean one. The polar coordinate system-based models are named as the corresponding complex-valued models with suffix “-polar”. Actually, the two-coordinate systems are related as follows:

(42) $$\begin{array}{c} w_{rj}=cos\left(\phi_{j}\right)r_{j}, \end{array}$$

(43) $$\begin{array}{c} w_{mj}=sin\left(\phi_{j}\right)r_{j}. \end{array}$$

Moreover, the claim in Li et al.’s paper that the radius amplitude corresponds to the classical word embedding with the lexical meaning is consistent with our assumption that the real embedding and imaginary embedding are a linear combination of sememes [25]. Therefore, our models’ performance is not closely related to the coordinate system chosen, which is compatible with the physical state assumption for a word. That is, the property of a physical state should be independent of coordination system chosen.

### 6.3. Attention Visualizations and Comparison

**Figure 4:**
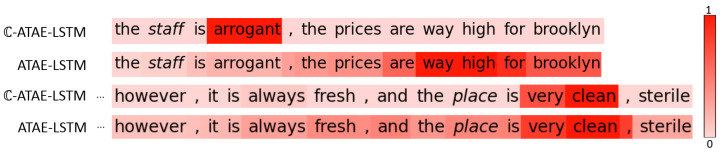
Attention visualization and comparison. The color of a word indicates the relative weight of attention for such word in each sentence, and the aspects are displayed in italic.

Compared with the real attention-based LSTM, our complex-valued one has better performance. To indicate the difference on attention weights between these two models, we visualize two selected sentences as shown in Figure 4. Figure 4 shows the degree of attention focusing on words with respect to a given aspect. The color density maps the importance degree of the weight. We can see that the attention weights of ATAE-LSTM have a wide spread over words, but those of C-ATAE-LSTM are more concentrated on the words relevant to the target aspects. “Arrogant” in “the staff is arrogant, …” has a dominant weight with respect to aspect “staff”, as well as “very clean” with respect to “place” in the second example. A possible explanation is that the real representation and imaginary representation interfere with each other, resulting in vanishing information irrelevant and hence boosting important information. Therefore, the important words “arrogant” and “very clean” are got enhanced and have dominant weights.

### 6.4. Case Study

In this section, we investigate some typical examples to explicitly show the advantage of our complex-valued models. Without the vanishing of generality, we mainly discuss cases for C-ATAE-LSTM and C-BERT. As shown in Table 5, the examples chosen are from the test set whose polarity labels are incorrectly inferred by the real-valued language models but correctly classified by the corresponding complex-valued models. All examples are chosen from Restaurant dataset. For C-ATAE-LSTM, two typical examples are shown. Sentence “the food was mediocre to be kind. the interior is small and average. the owners are a tag-team of unpleasantries. so rude and snotty.” has three aspect items, which are “food”, “interior” and “owners”. The whole sentence expresses a negative polarity on all three aspects, and the complex-valued model C-ATAE-LSTM can obtain the correct polarity. Sentence “not only is the food authentic, but the staff here are practically off-the-boat, they are young and hip and know what they are doing.” uses a collocation “not only … but …” with a negation word “not”. Our model can predict the correct polarity for both items “food” and “staff”. For C-BERT, the first sentence “although the restaurant itself is nice, i prefer not to go for the food.” does not include a direct polarity for aspect “food”. Especially, for “restaurant”, the polarity is quite positive, so the model needs to learn some logic underlying the sentence. The second sentence “the food is just okay, and it’s almost not worth going unless you’re getting the pialla, which is the only dish that’s really good.” also contains complex logic. The aspect term “dish” is easy to classify because of the obvious adjective word “good”. However, to correctly obtain the polarity for “pialla”, a model needs to learn the logic between “pialla” and “dish” and recognise that the negative polarity of words “not worth” is reversed by word “unless”.

## 7. Conclusions

In this paper, we have proposed quantum-inspired complex-valued language models for ABSA. The complexification of three typical real baselines is constructed. They are complex-valued LSTM, complex-valued attention-based LSTM, and complex-valued BERT/RoBERTa model. Experiments conducted on three benchmark datasets demonstrate the effectiveness of our complex-valued structure, which manifests that a complex-valued framework can improve a model’s performance in ABSA and shows that a complex-valued structure has a potential to benefit general neural networks. To demonstrate such generality, we can further investigate a complex-valued CNN model in the future, and construct models with more complicated structures. Our work also shows that quantum language model has not only a more fundamental mathematical and physical background, but also good performance. Through a detailed discussion, we show that the performance of our models is not closely relevant to the coordinate system chosen. This is consistent with the finding that a physical event is independent of the coordinate chosen.

Our current study is restricted to the ABSA field. However, such a complex-valued quantum language structure should have wider applications in various areas. Therefore, a further research direction may be to explore the application of quantum-inspired complexification in other NLP tasks. In addition, to manifest the influence of complex-valued representations throughout the whole pipeline, we could study a LSTM with built-in complex-valued cells and states.

## Figures and Tables

**Table 1:** Statistics of datasets.

Dataset	Positive		Neutral		Negative	
	Train	Test	Train	Test	Train	Test
Twitter	1561	173	3127	346	1560	173
Restaurant	2164	728	637	196	807	196
Laptop	994	341	464	169	870	128

**Table 2:** Results on Twitter dataset. The results with “*” are retrieved from [8]; those with “**” are retrieved from [15]; and those with “‖” are from [40]. The best performed values are in bold.

	Models	Twitter	
		Accuracy	F1
**RNN baselines**	TD-LSTM ${}^{*}$	0.7080	0.6900
**Ours**	C-LSTM	0.6922	0.6729
	C-ATAE-LSTM	0.7124	0.6913
**Ablation**	C-LSTM-random	0.6387	0.5881
	C-ATAE-LSTM-random	0.6503	0.6165
	C-LSTM-polar	0.6922	0.6698
	C-ATAE-LSTM-polar	**0.7153**	**0.6962**
**Transformer-based baselines**	BERT-SPC ${}^{**}$	0.7355	0.7214
	AEN-BERT ${}^{**}$	0.7471	0.7313
	RoBERTa-MLP ‖	0.7717	0.7620
**Ours**	C-BERT	0.7529	0.7380
	C-RoBERTa	**0.7745**	**0.7657**
**Ablation**	C-BERT-polar	0.7572	0.7455
	C-RoBERTa-polar	0.7702	0.7586

**Table 3:** Results on Restaurant dataset. The results with “♯” are retrieved from [9]; those with “*”, “**” or “‖” are retrieved from the same papers as Table 2; those with “†” are retrieved from the paper [16]. “/” means not reported. The best performed values are in bold.

	Models	Restaurant	
		Accuracy	F1
**RNN baselines**	LSTM ${}^{♯}$	0.7430	/
	TD-LSTM ${}^{*}$	0.7563	/
	ATAE-LSTM ${}^{♯}$	0.7720	/
**Ours**	C-LSTM	0.7643	0.6337
	C-ATAE-LSTM	**0.7866**	**0.6948**
**Ablation**	C-LSTM-random	0.6777	0.4120
	C-ATAE-LSTM-random	0.6804	0.4494
	C-LSTM-polar	0.7634	0.6283
	C-ATAE-LSTM-polar	**0.7866**	0.6775
**Transformer-based baselines**	BERT-SPC ${}^{**}$	0.8446	0.7698
	AEN-BERT ${}^{**}$	0.8312	0.7376
	BERT-ADA †	0.8714	0.8009
	RoBERTa-MLP ‖	0.8737	0.8096
	LCF-ATEPC †	**0.9018**	**0.8588**
**Ours**	C-BERT	0.867	0.8141
	C-RoBERTa	0.8848	0.8260
**Ablation**	C-BERT-polar	0.8616	0.7946
	C-RoBERTa-polar	0.8821	0.8295

**Table 4:** Results on Laptop dataset. “♯”, “*”, “**”, “‖”, “†” et al. indicate the same models in papers referred in Table 3. “/” means not reported. The best performed values are in bold.

	Models	Laptop	
		Accuracy	F1
**RNN baselines**	LSTM ${}^{♯}$	0.6650	/
	TD-LSTM ${}^{*}$	0.6813	/
	ATAE-LSTM ${}^{♯}$	0.6870	/
**Ours**	C-LSTM	0.6959	0.6280
	C-ATAE-LSTM	**0.7100**	**0.6591**
**Ablation**	C-LSTM-random	0.5831	0.4929
	C-ATAE-LSTM-random	0.5846	0.4952
	C-LSTM-polar	0.6944	0.6284
	C-ATAE-LSTM-polar	0.7085	0.6523
**Transformer-based baselines**	BERT-SPC ${}^{**}$	0.7899	0.7503
	AEN-BERT ${}^{**}$	0.7993	0.7631
	BERT-ADA †	0.8023	0.7577
	RoBERTa-MLP ‖	0.8378	0.8073
	LCF-ATEPC †	0.8302	0.7984
**Ours**	C-BERT	0.7994	0.7635
	C-RoBERTa	0.8480	0.8172
**Ablation**	C-BERT-polar	0.7993	0.7601
	C-RoBERTa-polar	**0.8495**	**0.8249**

**Table 5:** Case study examples. A. Example includes examples inferred correctly by C-ATAE-LSTM. B. Example includes examples inferred correctly by C-BERT. The aspects are displayed in italic. “Truth” is the ground truth for an example.

A. Example	Truth
the *food* was mediocre to be kind. the interior is small and average.	
the owners are a tag-team of unpleasantries. so rude and snotty.	0
the food was mediocre to be kind. the *interior* is small and average.	
the owners are a tag-team of unpleasantries. so rude and snotty.	0
not only is the *food* authentic, but the staff here are practically off-the	
-boat, they are young and hip and know what they are doing.	2
not only is the food authentic, but the *staff* here are practically off-the	
-boat, they are young and hip and know what they are doing.	2
**B. Example**	**Truth**
although the restaurant itself is nice, i prefer not to go for the *food*.	0
the food is just okay, and it’s almost not worth going unless you’re	
getting the *pialla*, which is the only dish that’s really good.	2
the food is just okay, and it’s almost not worth going unless you’re	
getting the pialla, which is the only *dish* that’s really good.	2

## Data Availability

We have used three datasets widely investigated in aspect-based sentiment classification task. Twitter dataset is a dataset gathered by Dong et al. [41]. Restaurant and Laptop are obtained from SemEval 2014 task 4 [4].
